# The Role of the Microbiome-Brain-Gut Axis in the Pathogenesis of Depressive Disorder

**DOI:** 10.3390/nu14091921

**Published:** 2022-05-04

**Authors:** Ewelina Młynarska, Joanna Gadzinowska, Julita Tokarek, Joanna Forycka, Aleksandra Szuman, Beata Franczyk, Jacek Rysz

**Affiliations:** Department of Nephrology, Hypertension and Family Medicine, Medical University of Lodz, ul. Żeromskiego 113, 90-549 Lodz, Poland; joanna.gadzinowska@stud.umed.lodz.pl (J.G.); julita.tokarek@stud.umed.lodz.pl (J.T.); joanna.forycka@stud.umed.lodz.pl (J.F.); aleksandra.szuman@stud.umed.lodz.pl (A.S.); bfranczyk-skora@wp.pl (B.F.); jacek.rysz@umed.lodz.pl (J.R.)

**Keywords:** microbiome-brain-gut axis, gut-brain axis, microbiota, depression, depressive disorder

## Abstract

The role of gut microbiota and its association with the central nervous system via the microbiome-brain-gut axis has been widely discussed in the literature. The aim of this review is to investigate the impact of gut microbiota on the development of depression and underlying molecular mechanisms. There are two possible pathways in which this interaction might occur. The first one suggests that depressive disorder could lead to dysbiosis and one of the causes may be the influence on the hypothalamic-pituitary-adrenal (HPA) axis. The second one considers if changes in the composition of gut microbiota might cause depressive disorder. The mechanisms that could be responsible for this interaction include the secretion of neurotransmitters, gut peptides and the activation of the immune system. However, current knowledge on this topic does not allow for us to state an unambiguous conclusion, and future studies that take into consideration more precise stress-measurement methods are needed to further explore direct mechanisms of the interaction between gut microbiota and mental health.

## 1. Introduction

### 1.1. What Is Metagenome?

It has long been known that the gut microbiota and metabolic processes in the human body interact with each other. Currently, microbiota refers to all microorganisms, i.e., bacteria, fungi, viruses and archaea, that inhabit the human body, while the term microbiome refers to the collection of their genomes, which is 100 times larger than the human genome [1], so that the host genome and microbiome constitute a common “metagenome.” Bacteria are the dominant population of the gut microbiome [2], accounting for up to 99% of the genes in the gut. Of these, we can distinguish the four most numerous and species-variable phyla, namely Bacteroidetes and Firmicutes [3] (representing about 90–99 percent), Proteobacteria and Actinobacteria [4]. For many years, it was thought that the role of the gut microbiome was primarily related to its activities in the gut, such as maintaining normal gut motility, digesting food, absorbing nutrients, and maintaining gut integrity [5].

### 1.2. How Are the Brain and the Gut Communicating?

Recent approaches to the subject, however, point to the microbiota’s ability to influence the central nervous system, specifically to promote a reciprocal bidirectional relationship between the brain and the gut. The brain and gut, through the microbiota, can influence each other’s functions via neuroendocrine, neuroimmune and sensory-neural molecular pathways. Moreover, both hypothalamic-pituitary-adrenal (HPA) axis and gut peptides might be involved in this communication system [6,7]. The connection between brain and gut microbiota can significantly impact stress, anxiety, cognition and neuropsychiatric disorders such as depression, bipolar disorder, schizophrenia, and anxiety [8,9]. The chronological order of the disturbances is not established, so it is not known exactly whether depression causes intestinal dysbiosis or whether it is changes in the composition of the microbiota that promote depression. On one hand, in an experiment conducted on rodents, depressive disorders developed in those who were transplanted with fecal samples from depressed patients. On the other hand, however, the rodents that exhibited depressive behavior and experienced stress showed alterations and reduced diversity in their gut microbiota [10]. This review paper will attempt to consider two hypotheses:

Does depressive disorder cause dysbiosis? Do alterations in gut microbiota lead to depression?

## 2. What Is the Gut Microbiota?

Gut microbiota is a complex and highly diverse community of trillions of microorganisms that live in the digestive tracts of humans and animals, including insects [11,12]. Microbiota are ten times more abundant than our somatic and germ line cells of the body [13]. The human gut microbiota consists of several types of microbes including bacteria, archaea, eukarya, viruses and parasites [14] that weigh approximately 1 kg and represents the first protection system of the gastrointestinal (GI) apparatus. The microenvironment of the gut favors the growth of bacteria from seven predominant divisions (Firmicutes, Bacteroidetes, Actinobacteria, Fusobacteria, Proteobacteria, Verrucomicrobia and Cyanobacteria) [15]. Among these, more than 90% of the total population is made up of the Bacteroidetes and Firmicutes [13]. The presence of the microbiota differs within the parts of the GI tract, from few micro-organisms in the stomach and small intestine, up to a concentration of approximately 10^12^ bacteria in the colon [16,17]. In humans, the gut microbiota has the biggest quantities of microorganisms, and the greatest number of species compared to other parts of the body [18]. 

Microbiota acquired at birth develop in parallel with the host and maintains its temporal stability and diversity through adulthood until death [19]. The gut microbiota forms an integral part of the human body [13] and plays a significant role in its normal functioning [11]. 

Though the gut microbiota is dynamic, it performs some basic immunological, metabolic, structural and neurological functions [13]. The metabolic role consists of the conversion of dietary elements into bioactive food components [8]. The gut microbiota scavenge about 10–30% of energy from the dietary fibers in the colon and the rest is excreted as feces [20]. Gut microbes possess an array of enzymes enabling the utilization of carbohydrates resistant to digestion by host digestive enzymes such as lignin, non-starch polysaccharides, resistant starch and oligosaccharides. Gut microbiota of the lower intestines ferments all of the dietary fibers, which results in the release of gases, short chain fatty acids (SCFAs), organic acids, and alcohols. SCFAs, the most prevalent being acetate, propionate and butyrate, meet about 10% of caloric demand of the host [21] and their main producers are *Roseburia* spp., *Eubacterium rectale*, *Faecalibacterium prausnitzii* and *Clostridium* spp. [22]. SCFAs also have promising anti-inflammatory and chemo-preventive properties [23]. According to the MEROPS database, gut microbiota have various peptidase and protease enzymes. *Clostridium* spp., *Bacteroides* spp. and *Lactobacillus* spp. are of special importance because of the diversity of possessed enzymes [24]. Some gut bacteria take part in the transformation of bile acids—their bile salt hydrolase deconjugates the unabsorbed bile salt and produces deoxycholate, ursodeoxycholate and lithocholate [8]. Gut microbiota executes its protective role by occupying intestinal surfaces and preventing the invasion of pathogenic microorganisms through creating a stable system. The epithelial cells of the mucosal barrier use the SCFAs as an energy source. Gut microbiota exhibit a significant impact on bone growth and development though SCFAs, regulation of calcium and phosphorus absorption from the diet and immunoregulation by the *Lactobacillus* spp. of the osteoclast-osteoblast mediated bone remodeling process [8]. Gut microbiota can control both the central and enteric nervous system through various mechanisms such as the production and expression of neurotransmitters and neurotrophic factors, modulating the enteric sensory afferents, metabolite production, immunoregulation of mucosa and maintaining the integrity of the intestinal barrier and tight junctions. 

There are several factors that can change the gut microbiota composition and function. Numerous studies have indicated that host genetics influences the composition of the gut microbiome [25]. Pattern recognition receptors modulate microbiome composition and the diseases associated with it. After birth, gut microbiota is shaped mainly by diet as the microbiome adjust to absorbed nutrients. Firstly, it is enriched in genes involved in the metabolism of breast milk’s oligosaccharides, whereas later in the ones associated with the digestion of polysaccharides and vitamins [26]. The method of feeding the newborn significantly influences the microbiome composition—breast-fed infants exhibit an overgrowth of Actinobacteria and an inhibition of Firmicutes and Proteobacteria, whereas formula-fed infants experience an increase of *Clostridium*, *Streptococci*, *Bacteroides* and Enterobacteriaceae [27]. Vegetarians exhibit the dominance of Firmicutes and Bacteroidetes [8]. The abundance of bile-tolerant species (*Bacteroides*, *Bilophila* and *Alistipes*) and suppression of Firmicutes have been correlated with a diet rich in protein and fats. Another factors significantly affecting the microbiota composition is age. The first year of age is considered to be the most important period of development. Taxonomic diversity is low at birth, but increases over time. Firmicutes and Bacteroidetes are dominating the adult gut microbiota, while the elderly exhibit a decrease in Bacteroidetes to Firmicutes ratio, a reduction in *Bifidobacterium*, amylolytic activity and SCFAs production and an abundance of Enterobacteriaceae [28]. Exercise increases the diversity of microflora by both internal and external factors such as overall healthy lifestyle, intrinsic adaptation to training, lower levels of inflammation, reduced morbidity and improved metabolic markers. Greater amounts of Firmicutes and a lower amount of Bacteroidetes were found in athletes as compared to non-athletes [8]. What is more, the antibiotics destroy both pathogenic and beneficial microbes causing dysbiosis—a disturbance of gut microbiota [29]. The kind of antibiotic and the length of the treatment are associated with the effect on gut microbiota. Moreover, studies have demonstrated an impact of smoking on microbiota composition, with the most significant impact observed in the oral cavity [30]. 

The disturbance of the gut microbiota population associated with the alteration of the microbial composition was proven to be related with diverse pathological conditions, i.e., inflammatory bowel diseases (IBD) [31], obesity and diabetes [32], allergy [33], autoimmune diseases [34] and cardiovascular disease [35]. Examples of changes in the composition of gut microbiota correlated with various diseases are shown in Table 1. 

The human gut microbiota has received considerable interest in recent years and our knowledge about the species and their potential applications is increasing with the increasing number of metagenomics studies [11].

## 3. What Is Major Depressive Disorder (MDD)?

Major Depressive Disorder (MDD) is a disease that affects more than 264 million people worldwide, approximately 800,000 of whom commit suicide annually [43]. However, there are many more people with MDD (>350 million), and, in addition, the number of undiagnosed people who suffer from subclinical depressive symptoms is estimated to be even higher [44,45]. It is worth noting that the prevalence of MDD over a 12-month period is almost identical when comparing high-income countries (5.5%) with low- and middle-income countries (5.9%). This indicates that MDD is neither a direct result of contemporary lifestyles in developed countries nor of poverty [46]. The diagnosis is made if the patient has the following symptoms: constantly depressed or depressive mood, feelings of guilt, anhedonia, feelings of worthlessness, lack of energy, trouble concentrating, changes in appetite, psychomotor slowing or agitation, insomnia and other sleep problems, or suicidal thoughts [47]. The phenomena associated with depression include, but are not limited to, deterioration in physical health and quality of life, increased unemployment, malfunctioning in the community, decreased productivity, and demand for improvements in the health care system [45]. 

Depression is associated the with abnormal function of the hypothalamic-pituitary-adrenal (HPA) axis [6]. Structural changes in the basal limbic system might also play a vital role in the pathogenesis of depressive disorder. This hypothesis is supported by both biochemical and histopathological findings [48]. Understanding the pathophysiology of depression is not easy because depressive syndromes are heterogeneous and their etiology is likely diverse [49]. Several mechanisms of this disease, as understood so far, suggest that there is a bidirectional influence between the gut microbiota and the central nervous system, including depression. The effects of depression on the gut microbiota are regulated by stress, changes in the release of neurotransmitters and other signaling molecules in the gut and dysregulation of the immune response [50].

## 4. Does Depressive Disorder Cause Dysbiosis?

### 4.1. Stress and Gastrointestinal Disorders

It was Hans Seyle who first hypothesized that stress can be a cause of multiple somatic disorders. According to his theory, an illness was considered to be a result of errors in the adaptation syndrome and therefore called stress the disease of adaptation [51]. This phenomenon was named the General Adaptation Syndrome (GAS) and described in 1956 in a book entitled “The Stress of Life” [52]. The biopsychosocial model in understanding the basis of gastrointestinal disorders was first proposed by Drossman in 1998. To date, gastrointestinal disease has been thought of primarily from a biomedical perspective, whereas Drossman’s work argues for the inclusion of stress in the exacerbation of gastroesophageal reflux disease and Crohn’s disease and central nervous system-regulated visceral hypersensitivity in the occurrence of gastrointestinal pain [53]. In experiments using animal models (rodents), it has been shown that although rodent microbiota is quantitatively different from human microbiota, both are qualitatively similar. Nevertheless, studies based on comparing microbiota in both species have many limitations [54]. Furthermore, it is easier to control the depressive stimuli acting on rodents in a laboratory setting and to turn individual stressors on or off than to carry out the same on humans, in which the presence and number of stressors is more complex [55].

### 4.2. Depressive State and Dysbiosis in Animal Models—Communication Routes

**Figure 1:**
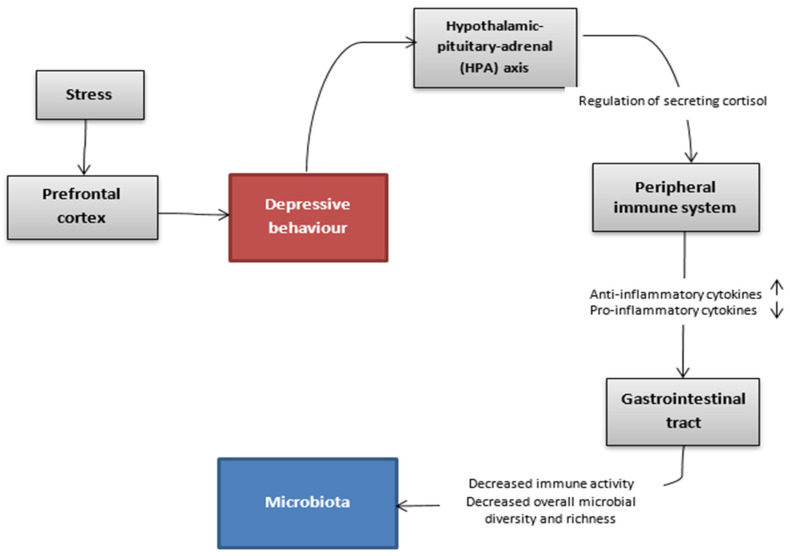
Hypothetic communication pathway between the brain and the gut microbiota in the depressive state. A depressed state triggers alterations in the microbiota via the hypothalamic-pituitary-adrenal axis and the immune system. This might lead to intestinal symptoms which can be further exacerbated by stress [10].

Therefore, we will first focus on demonstrating hypothetic cause-and-effect relationship between depressive stimuli and dysbiosis in animal models, which is presented in Figure 1. The use of bilateral olfactory bulbectomy in rodents causes severe dysfunction of the cortical-hippocampal-amygdala circuit that results in behavioral changes leading to anxiety–like and depressive behaviour. It has been shown that these parts of the central nervous system are probably also impaired in depressed patients [56]. Changes in rodent prefrontal cortex function following removal of the olfactory bulb have also been observed in humans with depression. The dysfunction of the prefrontal cortex in humans embraced hyperactivity in ventral-medial and hypoactivity in dorsolateral areas [57]. This surgical procedure induces behavioral changes in rodents that respond to chronic antidepressant treatment, thus mimicking the time course of treatment with antidepressants in a psychiatric ward [58]. A study conducted on mice after olfactory bulbectomy showed higher expression of corticotropin-releasing hormone (CRH) compared to controls, indicating the increased activity of the hypothalamic-pituitary-adrenal (HPA) axis. 

Kelly et al. conducted an experiment to verify the thesis that there is a bidirectional relationship between depressive states and microbiota composition. To conduct the study, researchers examined the saliva, serum, and fecal composition of 34 depressed patients and 33 healthy controls. Fecal samples from three patients with the most severe depression were combined and transplanted into 13 adult male rats that had previously been given antibiotics. In the study material, significantly increased levels of total cortisol output, IL-6, IL-8, TNF-α and CRP as well as a higher kynurenine/tryptophan ratio in depressed patients were detected. In stool samples from depressed patients, there was a reduction in the total number of species observed and low phylogenetic diversity. There were no significant differences in plasma lipopolysaccharide-binding protein or short-chain fatty acid levels, whereas depressive symptoms negatively correlated with daily dietary fiber intake. In an animal study, it was shown that rats with transplanted “depressed” microbiota exhibited anhedonia-like and anxiety-like behaviors compared to the control group. Plasma kynurenine concentration and kynurenine/tryptophan ratio were significantly increased in the depressed group, and plasma CRP concentration trended upward. Based on these results, the authors concluded that dysbiosis may play an important role in the pathogenesis of depression [59].

According to multiple studies, there is a shift in tryptophan metabolism from serotonin to the kynurenine pathway in depressed patients [60]. Tryptophan, the main precursor of the kynurenine pathway, is converted to kynurenine and then to other compounds such as anthranilic acid, kynurenic acid and 3-hydroxykynurenine. In the serotonin pathway, tryptophan is metabolized to 5-hydroxytryptophan, then to serotonin, then to 5-hydroxyindoleacetic acid [61]. Tryptophan levels are reduced in MDD, likely reflecting its relative importance in this disorder, particularly in the context of serotonin bioavailability. The decreased bioavailability of tryptophan is at least partially responsible for the decreased serotonin levels found in MDD. This decrease in tryptophan levels is most likely, at least in part, responsible for the decreased bioavailability of kynurenine found in MDD. Furthermore, we also observed an increase in the ratio of kynurenine to tryptophan in MDD, suggesting that the decrease in serotonin bioavailability, traditionally thought to be one of the bases of the monoamine hypothesis, is secondary not only to a decreased pool of tryptophan but also to a shift in tryptophan metabolism from serotonin toward kynurenine [60].

Hoban et al. investigated the behavioral and neurochemical consequences of chronic gut microbiota depletion during adulthood within rats. Adult male rats were administered antibiotics in order to deplete the intestinal microbiota, while a control group received no medications [62]. After six weeks, all rats underwent multiple tests assessing brain monoamine levels, microbiota composition, anxiety behaviors, depressive behaviors, colonic distension, and gene expression in the central nervous system, among others [63]. The authors found that antibiotic treatment caused significant depressive behavior, decreased 5-hydroxytryptamine (5-HT) levels and increased 5-hydroxyindoleacetic acid/5-hydroxytryptamine (5-HIAA/5-HT) turnover in the hippocampus. There was an increase in such parameters: tryptophan levels, norepinephrine levels in the striatum, levodopa (L-DOPA) and homovanillic acid (HVA) levels in the prefrontal cortex and hippocampus. Finally, the antibiotic-treated rats showed altered microbial diversity, with a significant decrease in Firmicutes and Bacteroidetes and an increase in Proteobacteria and Cyanobacteria. From these results, a distinct phenotype was identified, including depressive behavior and cognitive impairment, that was associated with antibiotic-induced microbiota depletion in rats during adulthood. Chronic exposure to antibiotics reduced the diversity and richness of the gut microbiota, which coincided with the occurrence of depression-like behaviors. Decreased levels of 5-HT and 5-HT/5-HIAA in the hippocampus and altered levels of L-DOPA and HVA showed the dysregulation of monoamine synthesis and degradation, indicating that dysbiosis may have profound effects on neurotransmitter systems [62]. 

### 4.3. Which Alterations Are Observed in the Depressed Brain

In patients with depressive disorders there are no alterations in the total brain volume, but there is a decrease in the volume of the hippocampus, prefrontal cortex, fronto-orbital cortex, anterior cingulate gyrus and subcortical structures—caudate nucleus, globus pallidus and putamen, suggesting atrophy or the loss of cells of these structures [64,65]. There is also a reduction in the proportion of gray matter compared to white matter within the prefrontal cortex [66]. Magnetic resonance imaging (MRI) studies have shown a reduction in hippocampal volume both in patients during a depressive episode and those with a history of such disorders in the past. These changes may result from the apoptosis of hippocampal cells or the inhibition of neurogenesis caused by glucocorticoid neurotoxicity [67]. The results of studies based on functional neuroimaging indicate a correlation between the applied treatment, severity of symptoms and the degree of morphological changes. In vivo resting-state functional magnetic resonance imaging (fMRI) studies have shown decreased connectivity between the prefrontal cortex, superior temporal gyrus and insular cortex, and increased connectivity between the amygdala and prefrontal cortex [68]. Microscopically, exposure to stress causes dendrite remodeling: a decrease in the density of dendritic spines in the prefrontal cortex and hippocampus, and an increase in their formation in the amygdala and nucleus accumbens [69,70]. Stress also affects neuronal survival and synaptic plasticity, which is mediated by brain-derived neurotrophic factor (BDNF). It has been shown that treatment with antidepressants and the use of non-pharmacological methods (electroshock, deep brain stimulation, transcranial magnetic stimulation) accelerate neuronal maturation, dendrite growth and maturation of dendritic spines and improve the survival of newly formed neurons [71]. 

Proteomic studies of the frontal cortex and anterior cingulate gyrus indicate the presence of abnormal cytoskeletal organization in depressed patients, which is associated with changes in the expression of, among others, glial fibrillary acidic protein (GFAP), tubulin isoforms or MAP proteins [72]. Suicide victims demonstrated the increased expression of mRNA and proteins such as TNF-alpha, IL-6 and IL1-beta in Brodmann’s area 10, suggesting the involvement of pro-inflammatory cytokines in the pathogenesis of psychiatric disorders [73]. Transmembrane TNF-alpha levels were also found to be increased in the dorsolateral prefrontal cortex, which is responsible for mood regulation. However, due to the absence of increase in other proinflammatory cytokines and their receptors, as well as an increase in neuronal integrity markers, it is presumed that the increased levels of transmembrane TNF-alpha are due to non-inflammatory causes [74]. In postmortem studies, serotonin transporter expression in the dorsolateral prefrontal cortex, ventral fronto-orbital cortex and brainstem is reduced in suicide subjects with a diagnosis of depression. Subtle structural changes in the monoaminergic nuclei of the brainstem, a major source of serotonin projection (dorsal raphe nucleus) and norepinephrine (locus ceruleus) to the cortex and increased number and density of tryptophan hydroxylase-responsive neurons in the dorsal raphe nucleus have been described in suicidal subjects with depression. Data on alterations in the number of pigmented neurons in the rostral locus ceruleus are inconsistent, but corticotropin-releasing hormone (CRH) immunoreactivity is increased in the locus ceruleus, dorsal and median raphe nuclei. Stereological studies of specific types of hypothalamic neurons showed increased numbers of arginine vasopressin– (AVP), oxytocin–, and CRH–neurons in the paraventricular nucleus, as well as increased CRH mRNA and corticoliberin neurons co-localized with AVP neurons. These data are consistent with reports of hypothalamic-pituitary axis activation in some depressed patients [68]. 

The symptoms of depression can be complex and vary widely between patients depending on the severity of the disease. Psychologically, patients presented continuous low mood or sadness, feeling hopeless and helpless, as well as having low self-esteem. Furthermore, feelings of worry, irritability and intolerance of others were reported by patients. Some of them confirmed the occurence of suicidal thoughts or thoughts of harming themselves [75]. 

In the context of physical symptoms, patients complained about changes in appetite or weight and, moreover, they moved or spoke more slowly than usual. Some of them were troubled by constipation and unexplained aches and pains, not to mention loss of libido and changes to the menstrual cycle in women [76]. Patients with depression experienced sleep disturbances and disruption of daytime rhythms. According to the European Research Society study (DEPRES II), two of the three most common symptoms reported during a current depressive episode were somatic in origin (fatigue/lack of energy/insomnia: 73%, interrupted sleep/decreased sleep: 63%) [77].

The social symptoms of depression included avoiding contact with friends and taking part in fewer social activities. Additionally, patients reported neglecting their hobbies and interests and having difficulties in work, school or family life.

### 4.4. Hypothalamic-Pituitary-Adrenal (HPA) Axis

Not only amongst mice, but also between humans, stress, depressive states and their allostatic response involve the sympathetic nervous system and the HPA axis. The activation of this system leads to the release of catecholamines from nerves and the adrenal medulla. This leads to the secretion of corticotropin from the hypothalamus and then boosts the adrenocorticotropin. It results in mediation of the release of cortisol from the adrenal cortex by corticotropin and adrenocorticotropin through a negative feedback loop and the inhibition of CRH and ACTH secretion. Corticotropin, in turn, stimulates adrenocorticotropin, which is the direct stimuli for the adrenal gland and its glucocorticoids [78]. Two types of glucocorticoid-activated receptors are responsible for this:

1. Mineralocorticoid receptors (MR) located mainly in the hippocampus

2. Glucocorticoid receptors (GR) in the hippocampus, hypothalamus and pituitary gland [79].

Research has long pointed to the association of abnormally activated hypothalamic-pituitary-adrenal (HPA) axis function with the illness of MDD, but it is difficult to clearly assess the utility of cortisol levels as an indicator of MDD pathophysiology. Certainly, higher cortisol levels as a stress response are found in patients with acute and severe forms of MDD [80]. According to numerous studies, the prefrontal cortex has neurons projecting to the hypothalamus, and therefore there is likely a pathway activated from the prefrontal cortex or hypothalamus that causes increased HPA axis activity [81]. 

### 4.5. Glucocorticoids and Suppression of the Inflammatory Response

Cortisol reduces the inflammatory response and prevents the body from developing an excessive immune response [82]. Cortisol increases the phagocytosis capacity of monocytes and macrophages, thereby promoting the removal of pathogens, cellular debris, foreign antigens and other harmful molecules. Acting through genomic and non-genomic mechanisms, this steroid hormone inhibits the production of pro-inflammatory cytokines, chemokines, and reduces the formation of reactive oxygen and nitrogen species. Glucocorticoids inhibit the production of proinflammatory cytokines, mainly those promoting differentiation to Th1 (IL-12 and interferon γ) and Th17 (IL-6 and IL-1), and furthermore enhance T cell migration to the bone marrow, spleen, lymph nodes, gastrointestinal lymphoid tissue and tonsils. The effect of this T-cell migration is to reduce the number of T-cells circulating systemically and to induce their apoptosis. According to studies, glucocorticoids inhibit the inflammatory response by stimulating the differentiation of regulatory T cells (Treg) and directly interact with the T cell receptor (TCR) signaling complex and inhibit its downstream transduction signaling pathways [83]. 

### 4.6. Chronic Stress Affects the Inflammatory Response

However, it is worth considering a condition not associated with the silencing of the inflammatory response by glucocorticosteroids, which is manifested by glucocorticoid receptor resistance (GCR) in a state of chronic stress which also occurs in Major Depressive Disorder. The model was proposed by Cohen et al. and, according to him, GCR is a result of long-term stressors, which in turn results in the production of more pro-inflammatory cytokines and a failure to silence inflammation [84]. In a meta-analysis of 82 case-control studies, Köhler et al. indicate a significant role for elevated concentrations of pro-inflammatory cytokines (i.e., IL-6, TNF-α, IL-10, the soluble IL-2 receptor, IL-13, IL-18, IL-12, the IL-1 receptor antagonist, the soluble TNF receptor 2, C-C chemokine ligand 2) in patients with MDD compared to non-depressed individuals [85]. Some studies highlight the important role of the inflammatory substrate in the pathophysiology of depression that should be given more consideration and should not be ignored [86].

### 4.7. Alterations of Intestinal Microbiota in Depressive State

Cortisol and glucocorticoids affect almost every type of immune cell due to the almost ubiquitous expression of GR, which is localized in the colonic epithelium [83]. Consequently, altered colonic motility was observed in mice after olfactory bulbectomy. Hence, increased colonic transit and a shift in the profile of the microbiota was noticed [87], which supports the hypothesis that the depressive state might promote alterations of intestinal microbiota through enhanced colonic activity [10]. Referring to the human model, a study on college students examined the effect of university stress on lactic acid bacteria activity. The number of lactic acid bacteria decreased significantly from a stress-free period to a highly stressful exam week and continued to decline [88]. Mice undergoing non-surgical methods of inducing a depressive state, exposed to a 10-day subchronic and mild social defeat stress (sCSDS), demonstrated changes in the microbiome as well as mice after olfactory bulbectomy [89,90]. Mice subjected to sCSDS had an increase in OTUs (Operational Taxonomic Units) belonging to the families Rikenellaceae, Desulfovibrionaceae and Lachnospiraceae, and showed a decrease in OTUs from the genera *Allobaculum* and *Mucispirillum* compared to the control group. However, it is important to remember that prolonged exposure to a stressor and changes in food intake, including increased appetite and thirst as a response to stress, may be integral components affecting the gut ecosystem. Changes in microbiota composition in depressed humans or stressed mice are found in Table 2.

## 5. Do Alterations in Gut Microbiota Lead to Depression?

**Figure 2:**
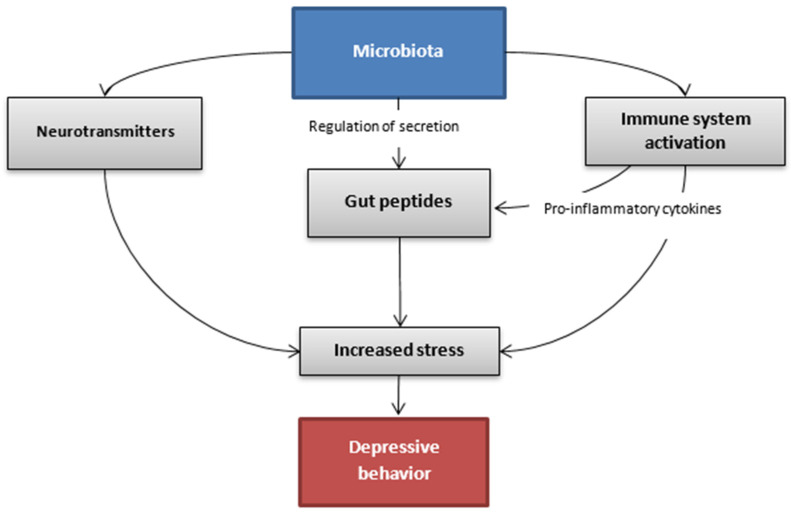
Hypothetic communication pathways between the gut microbiota and the brain in the depressive state.

The association between gut microbiota and depressive disorder has been the subject of many studies conducted in recent years. The complex mechanism, which might allow this bidirectional communication between intestines and the brain, is explained via the microbiota-gut-brain axis [99]. This pathway includes the immune, endocrine and autonomic system as well as molecules originating from the microbiota that take part in the regulation of these interactions (Figure 2). Alterations in gut microbiota are not considered to be the main factor that leads to depression. However, they are an important part. 

What we know is that rodents suffering from depression have a changed ratio in profitable bacteria such as *Bacteroides* and Firmicutes compared to healthy ones. According to the meta-analysis, bacteria from the families Veillonellaceae, Prevotellaceae and Sutterellaceae were less numerous in patients with MDD than in healthy controls, although Actinomycetaceae were more abundant in patients with MDD than in healthy controls. In addition, the same meta-analysis showed that patients with MDD had decreased levels of genus *Coprococcus*, *Faecalibacterium*, *Ruminococcus*, *Bifidobacterium* and *Escherichia* and increased levels of *Paraprevotella* [100]. 

Interestingly, study observations showed that probiotics including combination therapy with antidepressants had a large effect on depressive symptoms compared with the control group [101]. *Lactobacillus*-only trials showed that *Lactobacillus* have no clinical effects on depression based on the finding that *Lactobacillus*-only trials had a small, non-significant pooled effect in contrast to the significantly larger effects for other probiotic trials for depression [102].

Furthermore, the composition of intestinal microbiota might have an impact on the secretion of gut peptides and therefore regulate the endocrine activity of these molecules in the whole organism. As we already know, gut peptides regulate the endocrine activity and can communicate with the central nervous system. Their job is not only connected with food intake, but also with stress behaviors and reactions to such situations. The composition of intestinal microbiota might have an impact on intestinal barrier permeability and thus secreted gut peptides do not enter the braincells in the same way and efficacy what can implicate in different action of peptides in organism. That difference can result in altered behavior of decreased mood pursuing to depressive-like behavior [7]. 

### 5.1. What Are the Gut Peptides?

The group of gut peptides consists of over 20 molecules secreted by enteroendocrine cells (EECs) that perform many different signaling functions including endocrine and metabolic activity, and moreover present the ability to communicate with the central nervous system (CNS). The most important gut peptides include peptide YY (PYY), glucagon-like peptide (GLP-1), cholecystokinin (CCK), corticotropin-releasing factor (CRF), ghrelin and oxytocin (Table 3) [103]. 

### 5.2. How Does the Microbiota Interact with the Secretion of Gut Peptides?

Firstly, the changes in the composition of gut microbiota might lead to alterations in the intestinal barrier permeability through the interaction with endothelial tight junctions (TJs). It might cause an imbalance in the amount of gut peptides absorbed to the circulation and, furthermore, influence their function on the braincells [7,117]. Moreover, some Gram-negative bacterial genres present in the gut microbiota secrete an endotoxin, lipopolysaccharide (LPS), which promotes the activation of immune cascades and the production of pro-inflammatory cytokines. The pro-inflammatory phenotype associated with gut dysbiosis might be a trigger factor for the stress-induced inappropriate secretion of gut peptides [117]. Apart from the gut peptides, there are also specific molecules secreted by microorganisms, including metabolites and neurotransmitters (e.g., GABA, serotonin, tryptophan metabolites, catecholamines) that might penetrate to the bloodstream and act directly on receptors in the brain [99].

### 5.3. What Changes in the Composition of Gut Microbiota Might Cause Depressive Disorder?

The studies conducted both on human and animal subjects have suggested that there might be some differences in the composition of gut microbiota between healthy and depressed individuals. The strongest association refers to the Firmicutes/Bacteroidetes ratio [118,119]. Rodents with a higher amount of Bacteroidetes and a lower share of Firmicutes in their intestines had a tendency toward depressive-like behavior [119]. Moreover, mice subjected to chronic stress had decreased populations of *Bacteroides* and increased ones of *Clostridium* [94]. The association between fecal microbiota transplants from depressed subjects to healthy ones has also been the subject of research in different studies. For instance, the results showed that rats colonized with the microbiota from depressive-like individuals developed the symptoms of depressive behavior. However, no specific changes in the composition of microbiota that might be the cause of this phenomenon have been found [59]. Furthermore, several metanalyses have taken into consideration the effect of probiotic usage on mood [120,121,122]. Some of them proved that patients with symptoms of depression might benefit from this kind of supportive treatment (mostly using probiotics containing *Lactobacillus* and *Bifidobacterium* species) [123]. However, no profiling of gut microbiota was conducted on the participants before and after the use of probiotics and probiotics with different compositions of bacterial species that were used in the studies. Some of the studies did not find any effect of probiotics on depression [124]. These are the reasons why the use of probiotics in the treatment of depression still requires further research.

## 6. Conclusions

This section summarizes the information about how molecular mechanisms can affect the microbiome-gut-brain axis. It is widely known and has been deeply researched that stress (especially constant) is an indicator of gut microbiota alterations. Constant stress can impact the ratio of valuable bacteria in the human gut. There are more factors that influence communication pathways between gut microbiota and the brain.

It is said that the composition of intestinal microbiota might have an impact on gut peptides secretion and is responsible for balance in the endocrine system. Excessive excretion of pro-inflammatory factors (cytokines) has a role in gut microbiome changes because cytokines influence gut peptides, which are absorbed through the intestinal barrier of modified permeability. It then leads to the imbalance of the number of peptides delivered to the brain. It might be a trigger factor for changes in the microbiome, which could then cause depressive-like behavior. This is not to mention the specific molecules such as metabolites and neurotransmitters produced by microorganisms (LPS), which when secreted can instantly access the brain receptors. However, gut peptides and immune hypersensitivity caused by stress is just one factor of bidirectional influence on gut microbiota that could cause depression.

Some researchers claim that an abnormally activated HPA axis function (due to the high cortisol release) is the reason for MDD. The central nervous system and, more specifically, the prefrontal cortex dysfunction is known to be impaired amongst people suffering from MDD.

This review aimed to answer whether depressive-like behavior is an indicator of gut microbiota change or whether it is the other way round. As stated above, there are studies showing that the gut microbiota is altered because of stress. One such study has been carried out on students during a stressful exam period. On the other hand, people and rodents suffering from MDD have been shown to have gut dysbiosis and microorganism ratio imbalance (Bacteroidetes and Firmicutes). The healthy population does nott suffer from gut dysbiosis and microorganism ratio imbalance.

To conclude, it is nearly impossible to decide which molecular mechanism is more likely to explain the etiology of depression, although they both show how they affect organisms. It is unfeasible to measure stress intensity or cortisol levels in real life environments, and thus it is impossible to create constant, unmodified conditions in which the study could be conducted, unlike with rodents. Future studies could try to create an isolated environment where stress could be precisely measured.

The positive outcome of this scientific paper is that the most important mechanisms known for affecting the microbiome-brain-gut axis were gathered and discussed. This gives us an idea of how it possibly generates depressive-like behavior. Moreover, it also encourages us to focus more on this topic and to conduct further research. Future studies could possibly create new treatments for or contribute to the prevention of MDD.

## Figures and Tables

**Table 1:** Correlation of diseases with the changes in gut microbiota composition.

Disease	Paper	Increase	Decrease
Irritable bowel syndrome	Jeffery et al. (2012) [36]	Firmicutes especially *Clostridium*, *Ruminococcus* and *Dorea* ^a^	*Ruminococcus albus*, *Bacteroides fragilis*, *Bacteroides vulgatus* and *Ruminococcus callidus* ^a^
Inflammatory bowel disease (IBD)	Nishida et al. (2018) [37]	Mucolytic bacteria (*Ruminococcus gnavas, Ruminococcus torques*), sulfate-reducing bacteria (*Desulfovibrio*), pathogenic bacteria (adhesion/invasive *Escherichia coli*)	Firmicutes, SCFA-producing bacteria (*Clostridium* cluster IV, XIVa, XVII and *Faecalibacterium prausnitzzi*)
Obesity	Le Chatelier et al. (2013) [38]	*Porphyromonas, Campylobacter, Bacteroides, Staphylococcus, Parabacteroides, Dialister* and *Ruminococcus*	*Lactobacillus, Bifidobacterium, Faecalibacterium*, *Akkermansia*, *Methanobrevibacter* and *Coprococcus*
Insulin resistance and Diabetes mellitus type 2	Munoz-Garach et al. (2016) [39]	Firmicutes, *Lactobacillus gasseri, Streptococcus mutans, Escherichia coli*	Bacteroidetes, *Roseburia*, *Eubacterium halli, Faecalibacterium prauznitzi*
Hypertension	Dan et al. (2019) [40]	*Acetobacteroides, Alistipes, Bacteroides, Christensenella, Clostridium* sensu stricto, *Desulfovibrio, Parabacteroides*	*Acetobacteroides, Clostridium, Coprobacter, Enterococcus, Enterorhabdus, Lachnospiracea, Lactobacillus, Paraprevotella, Prevotella Romboutsia, Ruminococcus, Veillonella*
Asthma	O’Connor et al. (2018) [41]	*Bifidobacterium adolescentis*	*Staphylococcus aureus, Faecalibacterium prausnitzii* and *Clostridium*
Autistic spectrum disorder	Strati et al. (2017) [42]	*Collinsella, Corynebacterium, Dorea* and *Lactobacillus*	*Alistipes, Bilophila, Dialister, Parabacteroides* and *Veillonella*

^a^—selection of microbiota listed by this paper.

**Table 2:** Alterations in the intestinal microbial diversity observed in patients with depression and animals exposed to stress.

Phylum	Class	Order	Family	Genus	Model Organism	Population Shift
Actinobacteria	Actinobacteria	Coriobacteriales	Coriobacteriaceae	Unidentified genera	Mice	Increase [91]
Bacteroidetes	Bacteroidia	Bacteroidales	Rikenellaceae	Unidentified genera	Mice	Increase [90], Decrease [92]
Bacteroidetes	Bacteroidia	Bacteroidales	Porphyromonadaceae	*Odoribacter*	Mice	Increase [91]
Proteobacteria	Deltaproteobacteria	Desulfovibrionales	Desulfovibrionaceae	*Desulfovibrio*	Mice	Increase [90]
Proteobacteria	Alphaproteobacteria	Rhodobacterales	Hyphomonadaceae	*Ponticaulis*	Mice	Increase [93]
Firmicutes	Clostridia	Clostridiales	Lachnospiraceae	*Pseudobutyrivibrio*	Mice	Decrease [94]
Firmicutes	Clostridia	Clostridiales	Lachnospiraceae	*Coprococcus*	Mice	Decrease [94]
Firmicutes	Clostridia	Clostridiales	Lachnospiraceae	*Roseburia*	Mice	Increase [94]
Firmicutes	Clostridia	Clostridiales	Lachnospiraceae	*Dorea*	Mice	Decrease [94]
Firmicutes	Clostridia	Clostridiales	Peptostreptococcaceae	*Clostridium*	Mice	Increase [94]
Firmicutes	Clostridia	Clostridiales	Ruminococcaceae	*Oscillospira*	Mice	Decrease [92]
Firmicutes	Bacilli	Lactobacillales	Enterococcaceae	*Enterococcus*	Mice	Increase [92], Decrease [95]
Firmicutes	Bacilli	Lactobacillales	Lactobacillaceae	Unidentified genera	Mice	Decrease [93]
Firmicutes	Bacilli	Lactobacillales	Lactobacillaceae	*Lactobacillus*	Mice	Increase [92], Decrease [93,94]
Firmicutes	Erysipelotrichia	Erysipelotrichales	Erysiopelotrichaceae	*Allobaculum*	Mice	Decrease [90]
Deferribacteres	Deferribacteres	Deferribacterales	Deferribacteraceae	*Mucispirillum*	Mice	Decrease [90]
Bacteroidetes	Bacteroidia	Bacteroidales	Rikenellaceae	*Alistipes*	Mice, Human	Increase [91,96]
Bacteroidetes	Bacteroidia	Bacteroidales	Porphyromonadaceae	Unidentified genera	Mice, Human	Increase [96], Decrease [93,97]
Bacteroidetes	Bacteroidia	Bacteroidales	Porphyromonadaceae	*Parabacteroides*	Human, Mice	Increase [96], Decrease [93,94]
Firmicutes	Clostridia	Clostridiales	Lachnospiraceae	Unidentified genera	Human, Mice	Increase [90], Decrease [92,96,98]
Proteobacteria	Gammaproteobacteria	Enterobacteriales	Enterobacteriaceae	Unidentified genera	Human	Increase [96]
Actinobacteria	Actinobacteria	Coriobacteriales	Coriobacteriaceae	*Eggerthella*	Human	Increase [59]
Bacteroidetes	Bacteroidia	Bacteroidales	Bacteroidaceae	Unidentified genera	Human	Decrease [96]
Bacteroidetes	Bacteroidia	Bacteroidales	Bacteroidaceae	*Bacteroides*	Human	Decrease [96]
Bacteroidetes	Bacteroidia	Bacteroidales	Prevotellaceae	Unidentified genera	Human	Decrease [59,96]
Bacteroidetes	Bacteroidia	Bacteroidales	Prevotellaceae	*Paraprevotella*	Human	Increase [59]
Bacteroidetes	Bacteroidia	Bacteroidales	Prevotellaceae	*Prevotella*	Human	Decrease [59,96]
Firmicutes	Clostridia	Clostridiales	Lachnospiraceae	*Anaerofilum*	Human	Increase [59]
Firmicutes	Clostridia	Clostridiales	Lachnospiraceae	*Blautia*	Human	Increase [96]
Firmicutes	Clostridia	Clostridiales	Ruminococcaceae	*Ruminococcus*	Human	Decrease [96]
Firmicutes	Clostridia	Clostridiales	Clostridiaceae	*Faecalibacterium*	Human	Decrease [96]
Firmicutes	Clostridia	Thermoanaerobacterales	Thermoanaerobacteraceae	*Gelria*	Human	Increase [59]
Firmicutes	Erysipelotrichia	Erysipelotrichales	Erysiopelotrichaceae	Unidentified genera	Human	Decrease [96]
Firmicutes	Erysipelotrichia	Erysipelotrichales	Erysiopelotrichaceae	*Turicibacter*	Human	Increase [59]
Firmicutes	Erysipelotrichia	Erysipelotrichales	Erysipelotrichidae	*Holdemania*	Human	Increase [59]
Firmicutes	Negativicutes	Selenomonadales	Acidaminococcaceae	Unidentified genera	Human	Increase [96]
Firmicutes	Negativicutes	Veillonellales	Veillonellaceae	Unidentified genera	Human	Decrease [96]
Firmicutes	Negativicutes	Veillonellales	Veillonellaceae	*Dialister*	Human	Decrease [59,96]
Firmicutes	Negativicutes	Veillonellales	Veillonellaceae	*Megamonas*	Human	Increase [96]
Fusobacteria	Fusobacteriales	Fusobacteriaceae	Fusobacterium	Unidentified genera	Human	Increase [96]

**Table 3:** The most important gut peptides and their characteristics (HPA—hypothalamic-pituitary-adrenal).

*Gut Peptide*	Producing Cells	Releasing Factor	Peripheral Function	Central Function
**PYY** [104,105]	L-cells ^a^	food intake	inhibition of gastric emptying and intestinal motor activity	modulation of anxiety and stress-related disorders
**GLP**-1 [106,107]	L-cells ^a^	food intake	stimulation of insulin release and inhibition of glucagon secretion	modulation of the HPA axis and response to stress
**CCK** [108,109]	I-cells ^a^	food intake	suppression of appetite, gastric emptying, gallbladder contraction, pancreatic enzymes release	increased anxiety-like behavior
**CRF** [110,111,112,113]	effector neurons of hypothalamus and enterochromaffin cells of the colon	stress	inhibition of gastric emptying, stimulation of colonic motility and impairment of the intestinal epithelial barrier	increased anxiety and depressive disorder
**ghrelin** [114,115]	A-cells ^a^	starvation	increase of appetite and adipogenesis	modulation of stress response, anxiety and depressive disorder
**oxytocin** [116]	magnocellular neurons in hypothalamus	stress	facilitation of parturition and stimulation of lactation	reduced anxiety-like behavior and antidepressant effect

^a^—enteroendocrine cells present in the small intestine.

## Data Availability

The data used in this article are sourced from materials mentioned in the References section.
